# Role of Omentin in Obesity Paradox in Lung Cancer

**DOI:** 10.3390/cancers13020275

**Published:** 2021-01-13

**Authors:** Sheetal Parida, Sumit Siddharth, Dipali Sharma

**Affiliations:** Department of Oncology, Johns Hopkins University School of Medicine and the Sidney Kimmel Comprehensive Cancer Center at Johns Hopkins, Baltimore, MD 21231, USA; dsharma7@jhmi.edu

**Keywords:** lung cancer, obesity, omentin

## Abstract

**Simple Summary:**

Unlike other cancers, lung cancer risk is inversely associated with body mass index (BMI) with limited mechanistic understanding. Overweight and obese patients have been consistently found to respond better to therapy and show better survival. The adipose tissue—in addition to storing energy—secretes multiple unique cytokines or adipokines. Our in silico analysis reveals that a novel adipokine, omentin, is significantly and consistently downregulated in lung cancers compared to healthy lung tissue. Omentin was also found to be negatively correlated with important oncogenic transcription factors like ELK4, FOXA1 and FOXC1. Our study warrants further mechanistic studies on the role of omentin in lung cancers.

**Abstract:**

Lung cancer remains the second-most-common cancer worldwide and is associated with the highest number of cancer-related mortality. While tobacco smoking is the most important risk factor for lung cancer, many other lifestyles and occupational factors significantly contribute. Obesity is a growing global health concern and contributes to ~30% cancer-related mortality, but unlike other lifestyle diseases, lung cancer is negatively associated with obesity. We meta-analyzed multiple case-control studies confirming increased survival and better outcomes in overweight and obese lung cancer patients. Tumor heterogeneity analysis showed significant enrichment of adipocytes and preadipocytes in normal lungs compared to lung cancers. Interestingly, one of the understudied adipokine, omentin, was significantly and consistently lower in lung neoplasms compared to normal lungs. Omentin has been examined in relation to osteoarthritis, inflammatory bowel disease, cardiovascular diseases, diabetes, chronic liver disease, psoriasis and some other cancers. Aberrant expression of omentin has been reported in solid tumors; however, little is known about its role in lung cancer. We found omentin to be consistently downregulated in lung cancers, and it exhibited a negative correlation with important transcription factors FOXA1, EN1, FOXC1 and ELK4. We, therefore, suggest that omentin may serve as a prognostic factor in lung cancer and explain the “obesity paradox” in lung cancer.

## 1. Introduction

Despite declining trends, lung cancer remains one of the leading causes of cancer-related mortalities across the globe. Approximately 228,820 new cases of lung cancer will be diagnosed in 2020, while 135,720 individuals will succumb to lung cancer-related mortality in the US (https://www.cancer.org/content/dam/cancer-org/research/cancer-facts-and-statistics/annual-cancer-facts-and-figures/2020/cancer-facts-and-figures-2020.pdf). Overall, it accounts for one-fourth of all cancer-related mortalities [1]. While tobacco smoking is known to be the most important risk factor for the development of lung cancer across the globe, indoor accumulation of radon gas released from the soil is another significant risk factor in the US [2]. Occupational hazards like rubber manufacturing, paving, roofing, painting, chimney sweeping, secondhand smoke, asbestos, exposure to metals like chromium, cadmium, arsenic, certain organic chemicals, radiation, air pollution, domestic fuel usage and diesel exhaust also significantly increase the lifetime risk of lung cancers [2,3]. Additionally, pathogens like *Mycobacterium tuberculosis* have also been found to increase lung cancer risk [2]. However, unlike other lifestyle-related diseases, including multiple cancers, lung cancer is negatively associated with obesity [4,5,6,7]. A negative association between body mass index and lung cancer risk can be affected by multiple other factors, including waist–hip ratio (WHR), body muscle mass vs. fat mass, body fat distribution and level of physical activity. Large epidemiological studies and multiple lines of evidence clearly demonstrate an important role of adiposity on various cancers; however, the mechanistic understanding remains incomplete.

Obesity is a complex physiological state caused partly due to an imbalance in energy metabolism. Extensive research has shown that the adipose tissue is, in fact, a well-vascularized stroma rich tissue composed of fat cells or adipocytes along with preadipocytes, fibroblasts, endothelial cells, macrophages and smooth muscle cells [8]. Adipose tissue is further characterized, based on its function, into white adipose tissue (WAT), the primary energy-storing site or brown adipose tissue (BAT), a major site for thermogenesis and lipid oxidation. Plasticity of adipose tissue is much appreciated, but little information is available regarding multilocular beige adipocytes observed during WAT to BAT conversion and vice versa [9,10,11]. The most extensively researched fat tissue is the WAT since it forms the major fat depot in adults and has been found to be extremely important in health and disease. Two major WAT deposits in the body are the visceral fat surrounding major organs and the subcutaneous fat located under the skin. Increased visceral fat deposits can result in metabolic dysfunctions, including insulin resistance, type 2 diabetes, hypertension, hepatic steatosis and atherosclerosis; subcutaneous fat, on the other hand, is known to improve insulin sensitivity and decreased the risk of diabetes [10]. WAT is now recognized as a major endocrine organ secreting multiple cytokines/adipokines including leptin, adiponectin, resistin, PAI-1, VEGF, TNFα, IL6, autotaxin, visfatin, chimerin, apelin, MCP1, RBP4, vaspin, progranulin, FBP4, FBP5 and omentin [12,13,14,15,16,17]. The two most well-studied adipokines, leptin and adiponectin, are known to have opposite effects [18,19,20,21]. While leptin is known to be tumor promoting, adiponectin has been shown to be protective against cancer [22,23,24,25,26,27]. 

In breast cancer, leptin can induce stemness via JAK/STAT3 regulated fatty acid β-oxidation; augment invasion and metastasis by stimulating macrophage associated IL18 activating NFκB signaling in TAMs and PI3K/Akt signaling in tumor cells [28]. It is also known to induce EMT in breast cancer cell lines by inducing pyruvate kinase M2, consequently, PI3K/Akt-signaling [29]. Adiponectin, on the other hand, inhibits breast cancer progression via cytotoxic autophagy by modulating the STK11/LKB and AMPK-ULK1-signaling [21]. It also induces apoptotic cell death in breast cancer cells by inducing AMPK and inhibiting the PI3K/Akt survival pathway [30]. In addition to adipokines, the adipose tissue also secretes free fatty acids, which function as potent signaling molecules. The adipose tissue regulates cancer progression by shaping the tumor microenvironment where a complex network of growth factors, free fatty acids, adipokines, cytokines and matrix remodeling enzymes maintain constant communication between the tumor cells and stromal compartment influencing tumor cell proliferation, tumor vascularization, intra-tumor hypoxia, cell adhesion, migration and metastasis [16]. In addition to inducing local signaling pathways, adipokines are released into the circulation, initiating chronic systemic inflammation, thus affecting recruitment and activation of immune cells. In bidirectional communication, the tumor cells modify the peritumoral adipocytes by de-differentiating them into preadipocytes and/or reprogramming them to cancer-associated adipocytes (CAAs). The CAAs secrete free fatty acids and adipokines, which are then taken up by the cancer cells to meet increased energy requirements of proliferating tumors by increased fatty acid oxidation [31,32]. The CAAs exhibit decreased lipid content and adipocyte markers; they are reprogrammed to secrete pro-inflammatory cytokines, which recruit various classes of immune cells [33]. The CAAs can undergo further modifications to form cancer associated fibroblasts (CAFs) and induce various MMPs and somatomedins encouraging macrophage differentiation [31,34]. Most of the studies focusing on the positive correlation between obesity and cancer were conducted in breast cancer, while a few studies have focused on prostate cancer, endometrial cancer, colorectal cancer (CRC), hepatocellular carcinoma (HCC) and gastric cancers. Very little is known regarding the unique negative correlation presented by lung cancer and obesity. Our study aims at understanding the possible preventive role of adipose tissue in lung cancer using an in silico approach.

Scientific evidence clearly supports the dual role of adipose tissue in organ-specific cancers in a context-dependent manner. We hypothesized that a complex role of adipokines in modulating lung cancer microenvironment could potentially explain the obesity paradox in lung cancer. Here, we compared the gene expression profile of lung neoplasm to healthy normal lung tissue in large data sets and examined the possible role of adipokines, with a special focus on a lesser-known adipokine, omentin. Omentin, also known as intellectin (ITLN), is a newly discovered adipokine expressed in a number of tissues, including the heart, lungs and ovaries.

The most abundant source of secretory omentin is the visceral adipose tissue [35]. However, it is also known to be secreted by goblet cells of the epithelial lining of major visceral organs including the colon, heart and lungs and the mesothelial layer of the omentum [36,37,38]. Intriguingly, while omentin is produced by visceral fat, it is downregulated in obese states and related metabolic errors like insulin resistance and glucose intolerance [39]. In relation to other adipokines, it has been demonstrated to be positively associated with serum adiponectin levels and negatively associated with leptin levels. Omentin-1 (ITLN1) and omentin-2 (ITLN2) represent the two isoforms of omentin. Omentin-1 (ITLN1) is the main circulating omentin in plasma whose concentration in the synovial fluid of osteoarthritis patients is negatively associated with pain and physical disability [40]. Low plasma omentin concentrations are also found in patients with type 1 or type 2 diabetes mellitus [41,42]. In context of cancers, omentin has been shown to have differential effects; in ovarian cancer, it prevents progression and metastatic dissemination via interaction with lactotransferrin (LTF) [43,44,45]. It also enhances glucose uptake by adipocytes resulting in decreased energy available for the cancer cells to proliferate [45]. In pancreatic cancer, on the other hand, serum omentin levels were found to be significantly higher in patients compared to healthy controls and have been demonstrated to have a potential diagnostic significance [46]. Similarly, omentin overexpression was observed in prostate cancer [47] while it was significantly downregulated in renal cancer patients [48]. Furthermore, in CRC, omentin plasma levels were found to be positively associated with CRC risk in patients with BMI (body mass index) < 30 whereas, in patients with BMI ≥ 30, it did not show any correlation [49]. In all above-mentioned cancers, obesity is considered a leading risk factor except for prostate cancer, where the data are conflicting. While some studies have evaluated the involvement of omentin in multiple cancers [50] and other disease states [51], its association with lung cancer remains to be understood.

## 2. Results

### 2.1. Adiposity Decreases Lung Cancer Risk

The associations between obesity and lung cancer have been confirmed in multiple epidemiological studies over decades. We meta-analyzed two case-control studies, both pooled studies involving seven cohorts [52,53]. We used R package epitools to calculate relative risk (Algorithm 1). Consistent with earlier findings, higher BMI (>25) associated with a lower risk of lung cancer irrespective of subtype, gender and smoking status (Table 1). Lean individuals exhibited a relative risk (RR) of 1.32715 compared to 1 in obese individuals. In order to understand the molecular basis of this association between obesity and lung cancer, we took an in silico approach where we retrieved gene expression data from public databases (NCBI-GEO and EMBL-EBI) and analyzed them to explore microenvironment changes in lung cancer compared to healthy lungs.

### 2.2. The Lung Cancer Microenvironment

**Figure 1:**
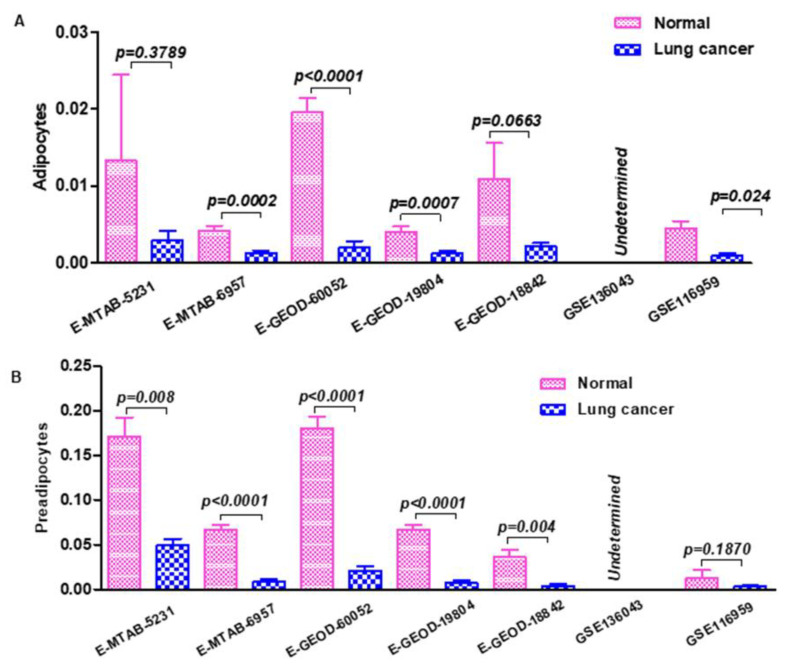
Tumor heterogeneity analysis to estimate adipocyte and preadipocyte population in lung cancer compared to normal lung tissue. (**A**) Fraction of adipocytes in normal healthy lungs vs. lung cancer in multiple data sets calculated using xCell. (**B**) Fraction of preadipocytes in normal healthy lungs vs. lung cancer in multiple data sets calculated using xCell.

We sought to decipher the protective mechanisms of body fat in lung cancer by examining the microenvironment of lung cancer. In this regard, we analyzed the tissue heterogeneity of lung neoplasms and healthy lungs in 7 gene expression datasets. We evaluated the 7 data sets for a 64-cell type signature and calculated the cell type enrichment score for each cell type using the web-based tool xCell (https://xcell.ucsf.edu/). The components evaluated include monocytes, CD8+ T-cells, NK cells, macrophages, endothelial cells, dendritic cells, neutrophils, erythrocytes, CD4+ naive T-cells, multipotent progenitors, smooth muscle cells, fibroblasts, epithelial cells, keratinocytes, chondrocytes, adipocytes, B-cells, CD4+ T-cells, CD8+ effector memory T-cells, common myeloid progenitors, granulocyte-macrophage progenitors, megakaryocyte–erythroid progenitors, regulatory t-cells, hematopoietic stem cells, plasma cells, CD4+ central memory T-cells, microvascular endothelial cells, CD4+ effector memory T-cells, memory B-cells, CD8+ central memory T-cells, naive B-cells, eosinophils, macrophages M1, myocytes, lymphatic endothelial cells, mesenchymal stem cells, macrophages M2, osteoblasts, activated dendritic cells, preadipocytes, melanocytes, skeletal muscle cells, CD4+ memory T-cells, megakaryocytes, pro B-cells, basophils, dendritic cells, astrocytes, plasmacytoid dendritic cells, pericytes, neurons, class-switched memory B-cells, hepatocytes, mesangial cells, immature dendritic cells, mast cells, type 2 T-helper cells, common lymphoid progenitors, platelets, type 1 T-helper cells, CD8+ naive T-cells, natural killer T-cells, sebocytes, and gamma delta T-cells. Surprisingly, while tissue composition was variable across samples and data sets, the most striking difference was the adiposity of the tissue sites. Irrespective of lung cancer subtypes, gender and smoking status, healthy normal control lungs as well as cancer- adjacent tissue was composed of significantly higher fractions of adipocytes and preadipocytes (Figure 1, Supplementary Materials Table S1), encouraging further investigation.

### 2.3. Differential Gene Expression in Lung Cancers

**Figure 2:**
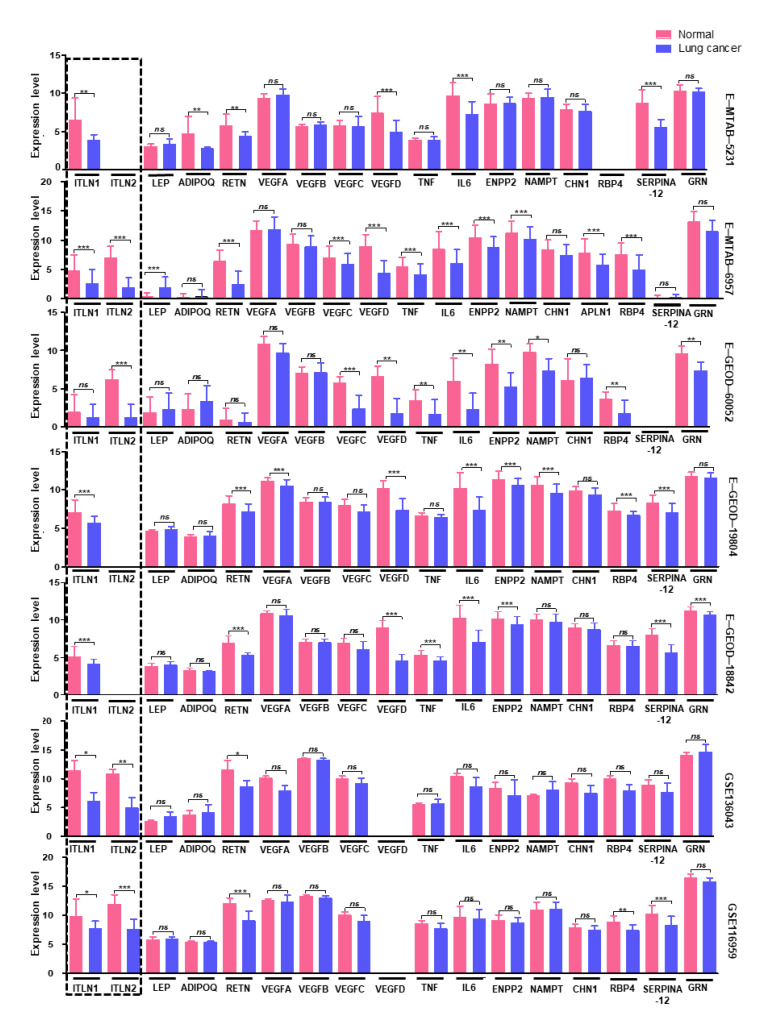
Expression levels of all adipokines in the indicated data sets. Omentin 1 (ITLN1) is significantly downregulated in lung cancer in 6 out of 7 data sets, and omentin 2 (ITLN2) is significantly downregulated in lung cancer in all 4 data sets in which it could be detected. In those 4 datasets, both ITLN1 and ITLN2 are downregulated in lung cancers. * *p* ≤ 0.01, ** *p* ≤ 0.001, *** *p* ≤ 0.0001.

The visceral fat is an endocrine organ secreting an array of chemokines, adipokines and free fatty acids, which initiate and propagate complex signaling pathways. Since the lung tumors were found to be depleted of adipose cells, to further understand the protective impact of the adipose rich microenvironment in lung cancer, we extensively analyzed the gene expression data sets by using the web-based platform iDEP.91 (http://bioinformatics.sdstate.edu/idep/). We examined the expression levels of adipokines leptin (*LEP*), adiponectin (*ADIPOQ*), resistin (*RETN*), *PAI-1*, *VEGF*, *TNFα*, *IL6*, autotaxin (*ENPP2*), visfatin (*NAMPT*), chimerin (*CHN1*), apelin (*APLN*), *MCP1*, *RBP4*, vaspin (*SERPINA12*), progranulin (*GRN*), *FBP4*, *FBP5* and omentin (*ITLN*) in the data sets. While no specific trends were observed in the expression levels of most of the cytokines, higher expression of omentin (*ITLN*) was consistently observed in normal lung tissue (Figure 2).

**Figure 3:**
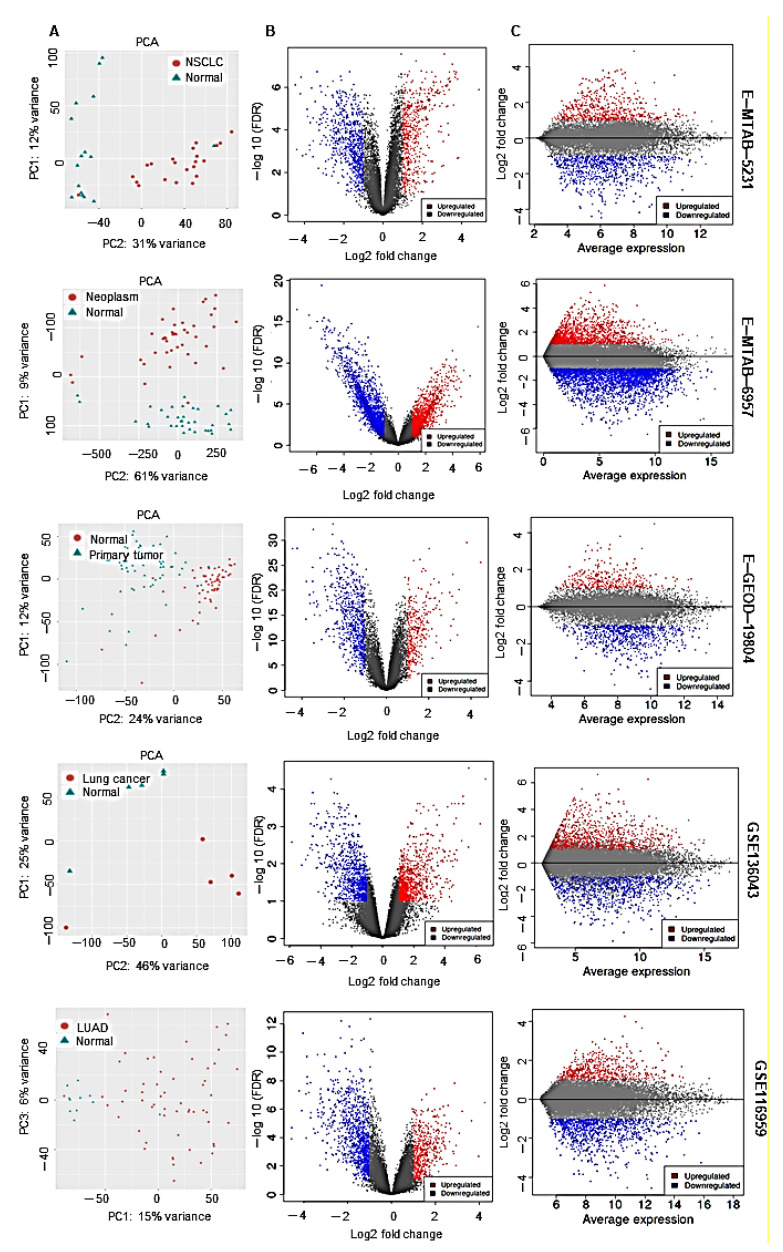
Differential gene expression analysis of data sets. (**A**) principal component analysis showing clustering of samples; (**B**) volcano plot showing differential expression of genes against false discovery rate between lung cancer and normal lungs; (**C**) MA plot (M: log ratio, A: Mean average) showing average expression of genes against fold change between lung cancer and normal lungs in respective data sets indicated to the right.

**Figure 4:**
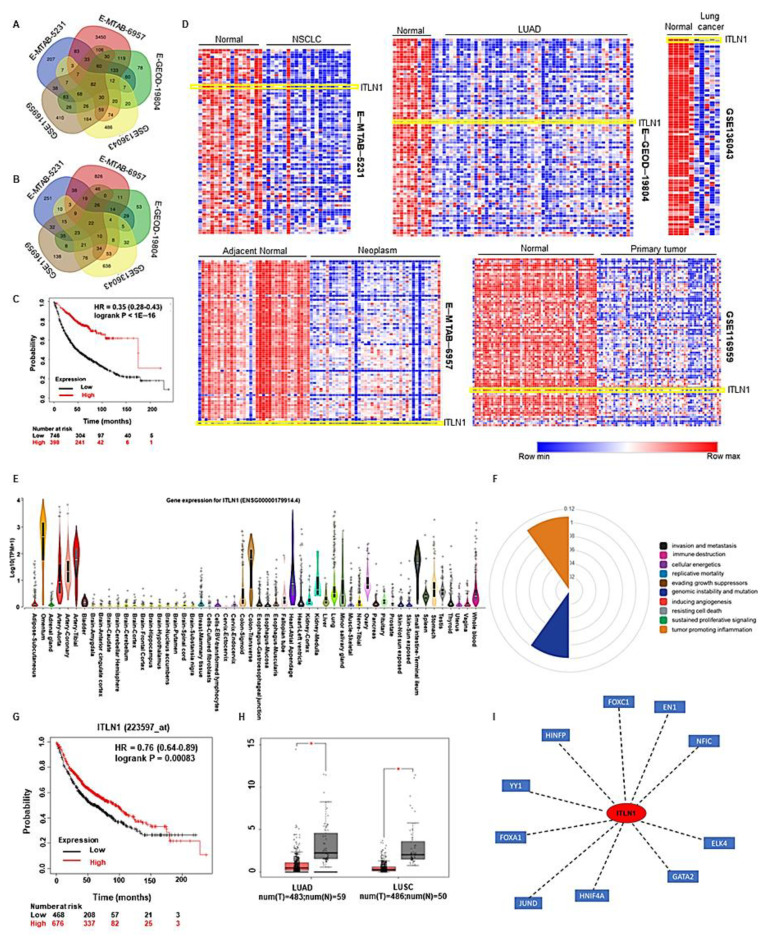
Overlapping differentially expressed genes (DEGs). (**A**) Venn diagram showing a number of differentially downregulated genes in lung cancer compared to the normal lung in all data sets. (**B**) Venn diagram showing a number of differentially upregulated genes in lung cancer compared to the normal lung in all data sets. (**C**) Kaplan Meier (KM) plot showing overall survival of lung cancer patients expressing high and low levels of the 82 DEGs. (**D**) Respective heat maps showing 82 commonly downregulated genes in all datasets. (**E**) Gene expression levels of ITLN1 in different normal tissues from the Genotype-Tissue expression (GTEx) portal. (**F**) Role of ITLN1 in cancer from cancer hallmarks analytics tool. (**G**) KM plot showing overall survival of lung cancer patients with high and low ITLN1 expression. Elevated ITLN1-associated with a better prognosis in lung cancer. (**H**) ITLN1 expression levels in different subtypes of lung cancer compared to normal lungs from Gene expression profiling interactive analysis (GEPIA) (**I**) ITLN1 associated transcription factors from NetworkAnalyst. LUAD: lung adenocarcinoma; LUSC: lung squamous cell carcinoma.

We evaluated the differentially expressed gene (DEGs), the enriched biological processes and pathways across the data sets using iDEP.91 (Figure 3; Supplementary Materials Figures S1 and S2; Supplementary Materials Tables S2–S4) as described in the methods section. We found 82 common DEGs downregulated while 22 DEGs upregulated in lung cancers in all the data sets (Figure 4A,B; Supplementary Materials Table S2). To find the overlapping DEGs, an adjusted p value cutoff of 0.05 was used. Among the differentially regulated biological processes, blood vessel development, vasculature development and circulatory system development were significantly downregulated in all data sets, while pathways related to mitotic division and nuclear division were consistently upregulated in lung cancers in all datasets. We did a survival analysis with the 82 commonly downregulated genes in lung cancer using KM plotter, and an extremely significant favorable outcome was associated with higher expression of the set of genes (H = 0.35, *p* < 1 × 10^−16^) in lung cancer patients (Figure 4C). Interestingly, *ITLN1* (omentin 1) was the only significantly downregulated adipokine in lung cancer compared to healthy lung tissue in all datasets (Figure 4D). We analyzed all the differentially regulated pathways, and surprisingly, pathways related to tube morphogenesis, vasculature development, etc., were found to be downregulated in lung cancers. On KEGG molecular pathway analysis, one pathway, “lipolysis in adipocytes,” was upregulated in all lung cancers compared to healthy lungs (Supplementary Materials Figure S3). Upregulation of lipolysis in adipocytes can potentially fuel the growth and progression of lung cancer cells.

### 2.4. Elevated ITLN1 Associates with Better Prognosis in lung Cancer

**Figure 5:**
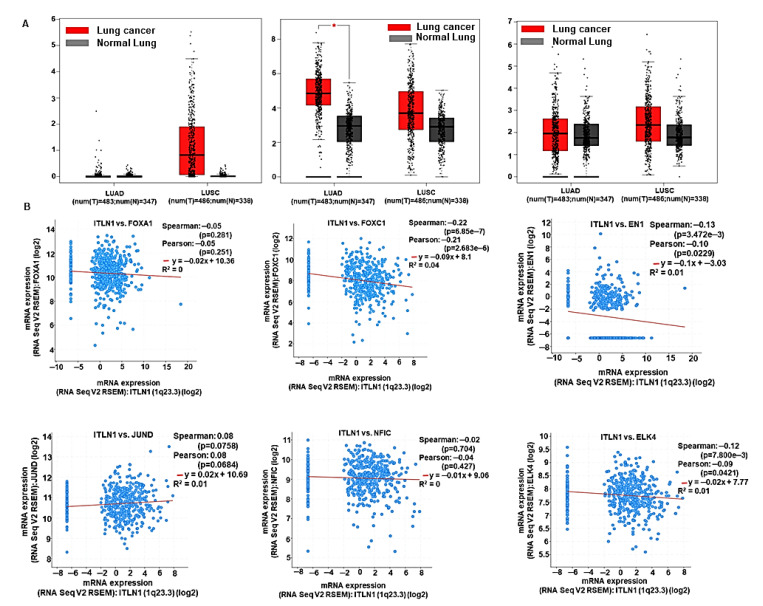
ITLN1 negatively correlates with oncogenic transcription factors in lung cancer. (**A**) Expression level of ITLN1 associated transcription factors in lung cancer compared to normal lungs. (**B**) Spearman correlation of ITLN1 with associated transcription factors from TCGA. LUAD: lung adenocarcinoma; LUSC: lung squamous cell carcinoma.

Next, we investigated the distribution of *ITLN1* in the human body using GTExPORTAL. Indeed, expression of *ITLN1* is high in the healthy lungs compared to other body sites (Figure 4E), which seems to be lost in lung cancers. We used the cancer hallmark analytics tool (https://chat.lionproject.net/) to investigate the potential role of *ITLN1* in cancer progression (Figure 4F). Though not much is known about it, *ITLN1* is thought to be important in driving genomic instability and mutation and cancer-promoting inflammation. Overall, survival in lung cancer patients expressing high levels of *ITLN1* was found to be significantly better compared to those expressing low levels of *ITLN1* (HR-0.76, *p* = 0.00083) (Figure 4G). We validated *ITLN1* expression in healthy lungs and lung cancers in a publicly accessible database, GEPIA (http://gepia.cancer-pku.cn/), which confirmed significant downregulation of *ITLN1* in lung cancer irrespective of subtype (Figure 4H). To attain a better mechanistic insight regarding the potential role of *ITLN1* in lung cancer, we examined its association with transcription factors using NetworkAnalyst (https://www.networkanalyst.ca/) and found 10 important associations; *FOXC1*, *EN1*, *NFIC*, *ELK4*, *GATA2*, *HNIF4A*, *JUND*, *FOXA1*, *YY1*, and *HINFP* (Figure 4I). Expression levels of *EN1*, *FOXA1* and *FOXC1*, were obtained from GEPIA (Figure 5A). Correlation of *ITLN1* expression with associated transcription factors was determined from TCGA database (TCGA Firehose Legacy) via cBioPortal. *ITLN1* expression negatively correlates with oncogenic transcription factors (*FOXA1*, *FOXC1*, *EN1*, and *ELK4*) (Figure 5B).

## 3. Discussion

Though still incompletely understood, obesity creates a state of chronic inflammation in the body priming the immune system and activating multiple cytokines. In most cases, visceral fat causes a hypoxic tumor microenvironment reprogramming cellular metabolism, thus enhancing their stress and wound healing responses; consequently, cancer cells are capable of proliferating more and surviving longer [54]. Excessive body fat disrupts the natural hormone balance of the body by causing insulin resistance and manipulating estrogen metabolism [55,56,57,58]. In line with these biological consequences of obesity on cancer cells, higher BMI and body fat evidently increase the risk and worsen patient outcomes in the breast, colorectal, endometrial, gall bladder, kidney, liver, ovarian, pancreatic, stomach and thyroid cancers, as well as multiple melanomas and meningiomas (https://www.cancer.gov/about-cancer/causes-prevention/risk/obesity/obesity-fact-sheet). Paradoxically, obesity or higher BMI (>25) has been consistently shown to be protective in lung cancers. Not only are leaner individuals at higher lifetime risk of lung cancer, but higher body weight also has been shown to be a positive prognostic factor in lung cancer.

The adipose tissue is a metabolically active endocrine organ secreting multiple hormones, growth factors, cytokines and free fatty acids capable of modifying the tumor microenvironment. The tumor microenvironment is a complex ecosystem composed of tumor cells, tumor vasculature, immune cells, adipocytes and other stroma components. Each component has a specific role in supporting tumor growth. The vasculature fuels the growing tumor mass while facilitating metastatic progression. First, the tumor secreted factors and cell-free DNA (cfDNA) are transported via the vasculature to distant organs, and then tumor cells are themselves carried through circulation and seeded in these distant niches. Similarly, cancer-associated fibroblasts (CAFs) protect the tumor cells from programmed cell death and promote migratory phenotype. The immune components, e.g., macrophages, play both tumor-promoting and inhibitory roles. The extracellular matrix (ECM) forms a scaffold for growing tumors. The adipose component is one of the main energy sources of tumors, and the adipocytes provide lipids and adipokines to the growing tumors. Adipocytes surrounding cancer cells undergo lipolysis to release free fatty acids (FFAs) and other lipids in the microenvironment in response to lipolytic signals from the tumor cells. In turn, tumor cells internalize FFAs to fuel lipid metabolic reprogramming and tumor growth. Intriguingly, from tumor heterogeneity analysis of multiple datasets, we found that lung tumors, irrespective of subtype, were depleted of adipocytes and preadipocytes. The adipose-derived factors induce multiple signaling cascades in nearby non-adipose cells via cell surface receptors, e.g., LEPR and ADIPOR for leptin and adiponectin, respectively. While leptin imparts a survival advantage to growing cancer cells, adiponectin acts quite the opposite.

We, therefore, sought to understand the consequence of adipocyte depletion in lung cancer progression. To that end, we investigated the expression of multiple adipokines. While none of the adipokines showed any specific trend, *ITLN1* and/or *ITLN2* genes encoding human omentin were consistently and significantly downregulated in lung cancers. On differential gene expression analysis, 82 significantly overlapping downregulated DEGs were identified, including *ITLN1*. This suggests a possible and important protective role of omentin in lung cancers. Omentin is a lesser-known adipokine, and mechanistic studies are necessary to understand its functions. *ITLN1* was found to interact with multiple important transcription factors. *FOXC1* is an inducer of epithelial to mesenchymal transition in cancers, thereby contributes to tissue invasion, metastasis and relapse in cancer [59]. *EN1* is an important developmental transcription factor that has been shown to be a biomarker in human salivary gland adenoid cystic carcinoma [60]. *NFIC* is a lesser-known transcription factor that has been shown to be a favorable prognostic marker in endometrial cancer [61]. *ELK4* is a transcription factor associated with the MAPK family and has been shown to be a prognostic indicator in prostate cancer [62]. *GATA2* is a regulator of embryonic stemness. *JUND* is an important proto-oncogene known to have varied roles ranging from maintenance of cells in a quiescent state to angiogenesis [63,64]. *FOXA1* is yet another transcription factor thought to be a determinant of cell fate [65,66]. It has been shown to play an important role in organogenesis and cancer progression. *YY1* is a transcriptional repressor known to have both cancer-promoting and cancer-inhibiting potential [67].

## 4. Materials and Methods

### 4.1. Data Acquisition and Quality Control

Two databases, EMBL-EBI gene expression atlas and NCBI-GEO were examined for gene expression by RNA sequencing or array for lung cancer versus normal lungs. To retrieve data from NCBI-GEO, the site was accessed on a web browser. From the All Resources drop-down menu, Gene Expression Omnibus (GEO) was accessed. Data sets were searched using the search term “lung cancer”. Studies comparing lung cancer and normal lung tissue gene expression were selected. Two datasets (accession number GSE136043 and GSE116959) were selected for analysis. Normalized read count data available as a series matrix file was downloaded. Sample information and sequencing/array platform information was retrieved. The data set was arranged as a gene expression matrix in MS Excel. Preprocessed data sets were then run through an integrated differential expression and pathway analysis (iDEP.91) pipeline. iDEP.91 is a web-based interface designed for fast, efficient and streamlined analysis of RNA sequencing and microarray data [68]. In order to retrieve data from the EMBL-EBI database, the website was accessed on the web browser. From the Services tab, the Gene Expression Atlas was accessed. From the Homo sapiens database, we browsed for gene expression data sets by RNA sequencing or array comparing lung cancer to healthy lung tissue. Five studies (Accession IDs E-MTAB-5231, E-GEOD-19804, E-MTAB-6957, E-GEOD-60052, E-GEOD-18,842) matched the criteria and were used for further analysis. The data sets were accessed, and expression values across all genes (FPKM file) and experimental design (TSV file) were downloaded. Since data available in EMBL-EBI is preprocessed, the FPK matrix was directly run through iDEP.91 pipeline. Data quality was checked with the preprocess function of iDEP.91. All searches were independent of smoking status, gender, age, race and lung cancer subtypes.

### 4.2. Tissue Heterogeneity Analysis

The tumor is a complex ecosystem of multiple cell types, each with specialized functions, which promote tumor cell proliferation and metastatic dissemination of cancer cells. In addition, multiple cell types interact to protect the developing tumor from the body’s natural defense mechanisms and secrete soluble factors, which help in establishing a metastatic niche in distant organs. To evaluate the heterogeneity of normal lung vs. lung cancer tissue, cell type enrichment analysis was performed on the above-mentioned data sets using the publicly accessible resource, xCell (https://xcell.ucsf.edu/). xCell is a novel web-based tool that combines the advantages of Gene set enrichment analysis with deconvolution approaches used by earlier methods to classify 64 cell types, including adaptive and innate immune components, hematopoietic progenitor cells and ECM components within the microenvironment based on gene expression data. To its credit, xCell has been validated using cytometry on a huge repertoire of cell types and transcriptomic data sets, making it more reliable than previously used techniques [69].

### 4.3. Gene Expression Analysis

For analysis of differential gene expression, we used a web-based interface, integrated Differential Expression and Pathway analysis (iDEP.91) [68], which takes advantage of the Shiny platform to integrate 63 R/Bioconductor packages, 2 web services, and comprehensive annotation and pathway databases for 220 plant and animal species simplifying the analysis of gene expression. The application can be used for exploratory data analysis in multiple ways, differential gene expression analysis, pathway analysis using KEGG as well as STRING-db and convenient visualization using superior ggplot2 graphics and interactive plots with Plotly. The workflow is completely reproducible, has been explained thoroughly in the website and also in the paper demonstrating analysis of two data sets [68]. Normalized gene expression data were uploaded as input file; systematic workflow was followed to visualize gene expression changes between normal lungs and lung cancer tissue. Principal component analysis was performed to visualize clustering of samples; significance of data distribution and fold changes were visualized using volcano and MA plots. Enriched biological processes and networks in sample subsets were generated from clustered enrichment analysis. Significantly altered molecular pathways were visualized using the KEGG application of iDEP.91. Differentially expressed genes (DEGs) in all sample sets were obtained. On differential gene expression analysis, we found downregulation of omentin encoded by ITLN1 or ITLN2 in all data sets. Since ITLN1 is the more ubiquitously expressed gene and not much is known about ITLN2, we selected the five datasets which had the most significant downregulation of ITLN1. Common DEGs in all datasets were retrieved by drawing a 5-way Venn diagram using the Bioinformatica and Evolutionary Genetics tool (http://bioinformatics.psb.ugent.be/webtools/Venn/), and common DEGs were used for further analysis. KM plotter [68], GEPIA (http://gepia.cancer-pku.cn/), NetworkAnalyst [70] and cancer hallmarks analytics tool (https://chat.lionproject.net/) were used to investigate genes of interest.

### 4.4. Statistical Analysis

For the metanalysis, odds ratio and risk ratio were calculated using the R package “epitools” (http://cran.r-project.org/web/packages/epitools/epitools.pdf) using the following script:
**Algorithm 1** 1:Library (epitools) 2:treatments <- c(“Control”, “Case”) 3:bmi <- c (“Obese”,”Lean”, “Overweight”) 4:dat <- matrix (c(279124,3900, 741,228, 13808, 600637,8909), nrow = 3, ncol = 2, byrow = TRUE) 5:dimnames (dat) <- list(“BMI” = bmi,”Treatments” = treatments) gender <- c (“male”,”female”) 6:dat2 <- matrix(c(395138,9128,463789,5721), nrow = 2, ncol = 2, byrow = TRUE) 7:dimnames (dat2) <- list(“Gender” = gender,”Treatments” = treatments) 8:smoke <- c (“never”,”current”,”former”) 9:dat3 <- matrix(c(410199,2254,176331,8008,272325,4557), nrow = 3, ncol = 2, byrow = TRUE) 10:dimnames(dat3) <- list (“Smoke” = smoke,”Treatments” = treatments) 11:oddsratio (dat) 12:riskratio (dat) 13:oddsratio (dat2) 14:riskratio (dat2) 15:oddsratio (dat3) 16:riskratio (dat3)

Epitools calculates risk ratio by “unconditional maximum-likelihood estimation (Wald), and small sample adjustment (small). Confidence intervals are calculated using normal approximation (Wald), and normal approximation with small sample adjustment (small), and bootstrap method (boot)”. Odds ratio is calculated by “median-unbiased estimation (mid-p), conditional maximum-likelihood estimation (Fisher), unconditional maximum-likelihood estimation (Wald), and small sample adjustment (small). Confidence intervals are calculated using exact methods (mid-p and Fisher), normal approximation (Wald), and normal approximation with small sample adjustment (small)”. For gene expression analysis, statistical packages in built-in iDEP.91 were used. All other statistics were performed using GraphPad Prism 5. Between-group comparisons were performed using Student’s t-test and one-way ANOVA. * *p* ≤ 0.01, ** *p* ≤ 0.001, *** *p* ≤ 0.0001.

## 5. Conclusions

In this in silico study, we aimed to address the “Obesity paradox in lung cancer” by investigating the lung cancer microenvironment. To our surprise, neither did we find any significant difference between stroma score, microenvironment score and immune score nor between specific cell types. However, the adipocytes and preadipocytes were strikingly higher in the normal tissue compared and found a single adipokine, omentin 1 (*ITLN1*), to be consistently and significantly underexpressed in lung cancers irrespective of gender, cancer subtype, age and smoking status. We investigated the biological pathways altered by the 82 common DEGs. However, we could only find associations with tumor angiogenesis and vasculogenesis because omentin is a relatively new and understudied adipokine and mechanistic understanding of its functions is still unclear. Our results warrant further investigation of the role of omentin in lung cancer. Moreover, additional omentin 1-independent signaling pathways between the adipocytes and cancer cells may be in place; further molecular studies are required for better understanding. The 82 identified DEGs were found to be an indicator of significantly better outcomes in lung cancer patients. We, therefore, propose that *ITLN1* could be an important prognostic marker in lung cancer and can also be the connecting link between increased BMI and improved lung cancer outcomes. Further mechanistic studies should be conducted to better understand the association between lung cancer and omentin1.

## Figures and Tables

**Table 1:** BMI (body mass index) and lung cancer risk. Irrespective of gender and smoking status, higher BMI correlates with lower lung cancer risk.

	Cases	Control	OR (95% CI)	*p* Value	RR (95% CI)	*p* Value
TOTAL (1,648,786)	27,617	1,620,989				
BMI						
Lean (*n* = 755,036) BMI < 25	13,808	741,228	1.3332	<0.0001	1.327155	<0.0001
Overweight (*n* = 609,546) 25 ≤ BMI < 30	8909	600,637	1.06155	<0.0001	1.060672	<0.0001
Obese (283,024) BMI ≥ 30	3900	279,124	1	NA	1	NA
GENDER						
Male (*n* = 404,266)	9128	395,138	1	NA	1	NA
Female (*n* = 469,510)	5721	463,789	0.533985	<0.0001	0.5395681	<0.0001
SMOKING STATUS						
Current (*n* = 184,339)	8008	176,331	8.26438	<0.0001	7.94927	<0.0001
Former (*n*=276,882)	4557	272,325	3.0451	<0.0001	3.01165	<0.0001
Never (*n* = 412,453)	2254	410,199	1	NA	1	NA

## Data Availability

All data sets used have been retrieved from NCBI-GEO and EMBL-EBI data bases. Accession numbers of all data sets used have been included in materials and methods section.

## Supplementary Materials

The following are available online at https://www.mdpi.com/2072-6694/13/2/275/s1, Figure S1: Differentially enriched biological processes in lung cancer compared to normal lung tissue, Figure S2: K means clustered heatmaps and corresponding visualization, Figure S3: Regulation of lipolysis in adipocytes enriched in lung cancers compared to healthy lung tissue, Table S1: heterogeneity analysis of all datasets, Table S2: DEGs lung cancer vs. Normal lungs, Table S3: Kmean enriched clusters, Table S4: Enriched biological processes.

## Abbreviations

WHR	Waist–hip ratio
BMI	Body mass index
WAT	White adipose tissue
BAT	Brown adipose tissue
PAI1	Plasminogen activator inhibitor 1
IL	Interleukin
VEGF	Vascular endothelial growth factor
TNF	Tumor necrosis factor
MCP1	Monocyte chemoattractant protein 1
RBP	Retinol-binding protein
CRC	Colorectal cancer
HCC	Hepatocellular cancer
CAF	Cancer-associated fibroblasts
CAA	Cancer-associated adipocytes
MMP	Matrix metalloproteinase
NFkB	Nuclear factor-kappa B
TAM	Tumor-associated macrophages
ITLN	Intelectin
EMBL-EBI	European Molecular Biology Laboratory-European Bioinformatics Institute
NCBI-GEO	National Center for Biotechnology Information-Gene Expression Omnibus
cfDNA	Cell-free DNA
ECM	Extracellular
RR	Relative risk
LUAD	lung adenocarcinoma
LUSC	lung squamous cell carcinoma
